# Prediction of Adolescents’ Fluid Intelligence Scores based on Deep Learning with Reconstruction Regularization

**DOI:** 10.21203/rs.3.rs-4482953/v1

**Published:** 2024-06-18

**Authors:** TingQian Cao, Xiang Liu, Jiawei Luo, Yuqiang Wang, Shixin Huang

**Affiliations:** West China Hospital of Sichuan University; Sichuan University; Sichuan University; West China Hospital of Sichuan University; The People’s Hospital of Yubei District of Chongqing city

**Keywords:** Fluid Intelligence, Autoencoder, Deep Learning, Structural MRI, Shapley Value

## Abstract

**Objective:**

The aim of this study was to develop a predictive model for uncorrected/actual fluid intelligence scores in 9–10 year old children using magnetic resonance T1-weighted imaging. Explore the predictive performance of an autoencoder model based on reconstruction regularization for fluid intelligence in adolescents.

**Methods:**

We collected actual fluid intelligence scores and T1-weighted MRIs of 11,534 adolescents who completed baseline tasks from ABCD Data Release 3.0. A total of 148 ROIs were selected and 604 features were proposed by FreeSurfer segmentation. The training and testing sets were divided in a ratio of 7:3. To predict fluid intelligence scores, we used AE, MLP and classic machine learning models, and compared their performance on the test set. In addition, we explored their performance across gender subpopulations. Moreover, we evaluated the importance of features using the SHapley Additive Explain method. Results: The proposed model achieves optimal performance on the test set for predicting actual fluid intelligence scores (PCC = 0.209 ± 0.02, MSE = 105.212 ± 2.53). Results show that autoencoders with refactoring regularization are significantly more effective than MLPs and classical machine learning models. In addition, all models performed better on female adolescents than on male adolescents. Further analysis of relevant characteristics in different populations revealed that this may be related to gender differences in underlying fluid intelligence mechanisms.

**Conclusions:**

We construct a weak but stable correlation between brain structural features and raw fluid intelligence using autoencoders. Future research may need to explore ensemble regression strategies utilizing multiple machine learning algorithms on multimodal data in order to improve the predictive performance of fluid intelligence based on neuroimaging features.

## 1 Introduction

The early understanding of children’s cognitive development can lead to improved health outcomes throughout adolescence. It is therefore crucial to identify the neural mechanisms underlying general intelligence. Fluid intelligence (Gf) refers to the ability to think logically and solve problems in novel situations, independent of acquired knowledge(Supekar, Swigart et al. 2013). According to general consensus, fluid intelligence peaks in late adolescence and declines thereafter. Therefore, its quantification and accurate prediction is essential for adolescents because it forecasts their creative achievement, academic performance, employment prospects, socioeconomic status, etc. magnetic resonance imaging (MRI) images of the brain’s structural and functional architecture are powerful tools for predicting fluid intelligence. The ABCD dataset provides MRI images and data on a large number of adolescent participants to accurately predict fluid intelligence scores. In the past, fluid intelligence has been studied in order to identify the mechanisms that underlie cognitive abilities. It has been shown that brain volume is strongly correlated with intelligence, and that this effect can be large(Gignac and Bates 2017). Linking brain structure to function at the neural level and at the level of phenotypic expression is a continuing challenge in neuroscience. Despite new neuroimaging methods, such as functional magnetic resonance imaging (fMRI) and diffusion tensor tractography, it remains challenging to link fundamental structural properties with complex behavioral expressions(Cole, Yarkoni et al. 2012). Researchers have used T1-weighted MRI to correlate brain structure with autism, Alzheimer’s disease, and Parkinson’s disease(Wang, Wee et al. 2015, Pominova, Kuzina et al. 2019, Sämann, Iglesias et al. 2022). There are, however, few structural MRI studies investigating more subtle differences in routine brain function, despite these abnormalities often being associated with gross differences in brain organization. Frontoparietal connectivity properties and other brain traits have been associated with fluid intelligence(Pohl, Thompson et al. 2019). Recently, structural information in MRI has been found to correlate with fluid intelligence(Cole, Yarkoni et al. 2012). A machine learning approach is described in reference(Kao, Zhang et al. 2019) for predicting fluid intelligence from brain MRI data. In general, fluid intelligence scores are predicted using existing computer-aided tools and a machine learning model trained on the extracted features(Pölsterl, Gutiérrez-Becker et al. 2019). In recent years, deep learning methods have emerged as state-of-the-art solutions to many problems across multiple domains, including natural language processing, bioinformatics, and medical imaging(Ahmad, Eckert et al. 2018, Rajkomar, Dean et al. 2019). As a deep learning model, autoencoders(Kramer 1991, Yang, Xu et al. 2021) are used to learn efficient encodings of inputs and have become effective feature extraction tools. In this study, we propose an autoencoder approach to predict fluid intelligence scores of adolescents by analyzing ROI shape features.

## 2 Materials and Methods

**Figure 1:**
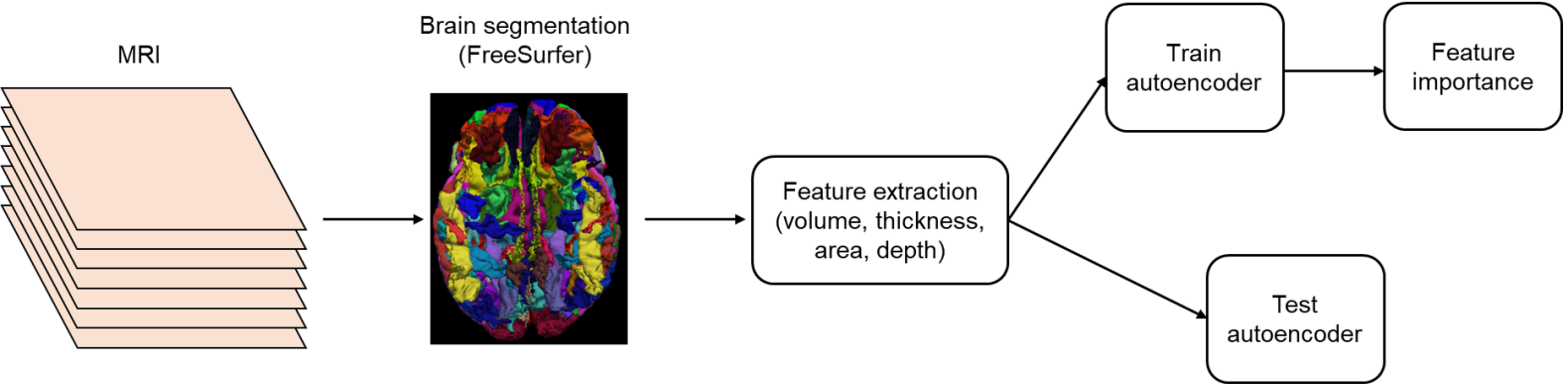
Overview of Our Proposed Pipeline for Predicting Fluid Intelligence from T1-Weighted MRI Scans based on Features in FreeSurfer.

Figure 1 illustrates our pipeline for predicting fluid intelligence based on T1-weighted MRI scans. The scans were performed in accordance with the protocol of the Adolescent Brain Cognitive Development (ABCD) study. To quantify brain morphometry, by assigning 74 neuroanatomical labels(Fischl, Salat et al. 2002) to each voxel for FreeSurfer segmentation, volumetric information for tissue classes including ventricles and subcortical grey and white matter structures can be obtained. We used the mean value of each region of interest (ROI), which was defined by the Destrieux(Desikan, Ségonne et al. 2006) atlas, divided into a total of 148 ROIs left and right, and one metric each for the left hemisphere, right hemisphere, and whole brain. We therefore extracted a total of 604 volume, area, depth and thickness measurements, all of which were produced by FreeSurfer. These brain measurements form the input to our deep autoencoders. Finally, we evaluate the feature importance of the final model using the recently proposed SHapley Additive explanation (SHAP)(Lundberg and Lee 2017).

### 2.1 Dataset and participants

In this study, data were obtained from ABCD Data Release 3.0 (https://nda.nih.gov/abcd). More than 11,000 adolescents aged 9–10 from 21 research centers across the country participated in the ABCD Study, a longitudinal study of brain, behavioral, and child health in the United States(Fan, Marshall et al. 2021). Written and oral informed consent was obtained from parents and children, respectively. Further information is available on the ABCD website (https://abcdstudy.org) and elsewhere. The neuroimaging and demographic data of 11,534 adolescents were screened according to whether they completed the baseline tasks. Fluid intelligence scores recorded in the ABCD study were measured using the NIH Toolbox Neurocognition battery.

### 2.2 Data preprocessing

All measurements were normalized while accounting for outliers by subtracting the median and dividing by the range between the 5% and 95% percentiles. In this way, we reduce the impact of outliers while still obtaining approximately centered features with equal scale. Then, all samples were divided into a training set and a test set according to a 7:3 ratio. Lastly, the fluid intelligence scores from the training data were standardized to a zero mean and unit variance; the same transformation was carried out on the validation and test data.

### 2.3 Model

**Figure 2:**
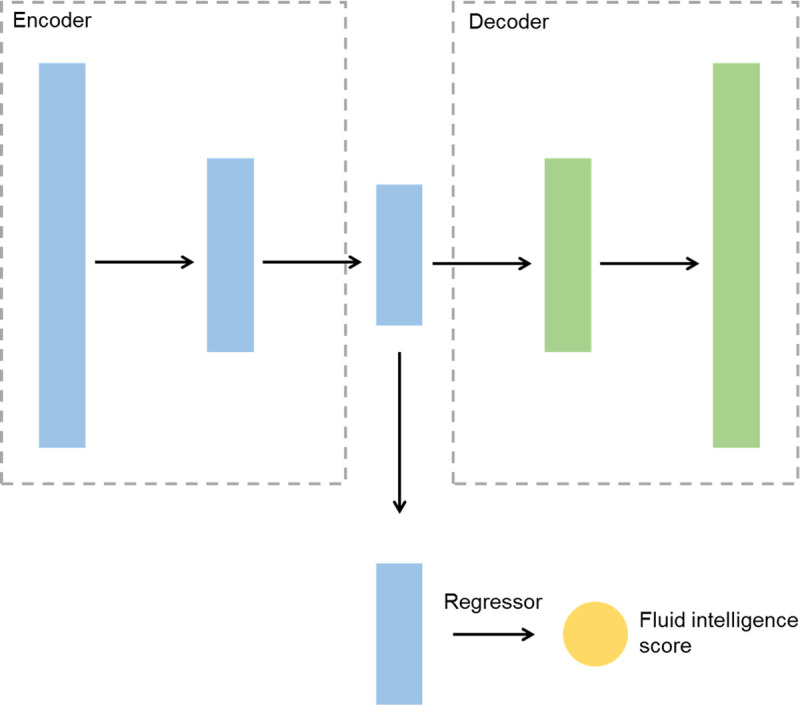
Illustration of the Autoencoder’s Workflow.

Autoencoder consists of two components: decoder and encoder. 𝒳 is the space of decoded messages and Z is the space of encoded messages. Both $𝒳={\mathbb{R}}^{m}$ and $Ƶ={\mathbb{R}}^{n}$ belong to Euclidean spaces, where m and n are the dimensions of the input feature vector and hidden vector, respectively. The encoder and decoder are denoted *E*_*φ*_ and *D*_*θ*_, respectively, where, *θ* and *φ* are the parameters to be learned. For any *x* ∈ *χ*, we note *z* = *E*_*φ*_ (*x*) as the encoding. And for any $z\in Ƶ,x^{\prime }=D_{\theta }(z)$ is noted as the decoded information. As described in Fig. 2, both the encoder and decoder are modeled as multilayer perceptron. Additionally, we established an additional regression branch for predicting fluid intelligence scores. For this branch, the encoded hidden vector is used as an input, and the predicted fluid intelligence score is used as an output. We denote this branch network as *y*′ = *f*_*ψ*_ (*z*), where *ψ* is the parameter to be learned and *y*′ is the predicted value. The motivation for our research is to optimize the process of reconstructing the input features in order to obtain an effective representation of the original features, which is the effective information that has been left after eliminating redundant information. The remaining valid information was used to predict fluid intelligence scores.

To prevent the model from overfitting, we imposed a regularization term of the L2-norm on all parameters. It is worth noting that *f* and *D* share the output of the encoder. According to the theory of multi-task machine learning, through the joint learning of multiple related tasks and sharing the representation of the hidden layer, a single task can help other tasks learn better feature representations, thereby improving the performance of the model for a single task. Finally, the objective function of the whole model is:

#(1) $$\underset{\theta ,\varphi ,\psi }{\text{argmin}}L(\theta ,\varphi ,\psi )={𝔼}_{x\sim p(x)}\left[d\left(x,D_{\theta }\left(E_{\varphi }(x)\right)\right)\right]+{𝔼}_{x\sim p(x)}\left[d\left(y,f_{\psi }\left(E_{\varphi }(x)\right)\right)\right]+\lambda_{\theta }∥\theta {∥}_{2}^{2}+\lambda_{\varphi }∥\varphi {∥}_{2}^{2}+\lambda_{\psi }∥\psi {∥}_{2}^{2}$$

where, $d\left(x,x^{\prime }\right)={\left\|x-x^{\prime }\right\|}_{2}^{2}$ is the mean square loss (MSE) function. λ_*θ*_, λ_*φ*_ and λ_*ψ*_ are hyperparameters that control the degree of regularization.

### 2.4 Feature importance

Although deep learning models exhibit the potential to solve a wide range of prediction tasks, their black-box nature often prohibits their application in clinical settings(Ribeiro, Singh et al. 2016, Ahmad, Eckert et al. 2018). To alleviate the problem of black-box predictions, we applied the Shapley additive explain (SHAP) algorithm to predictive models. A Shapley value is a model-independent way of expressing the influence of features on a particular prediction. It comes from cooperative game theory. Based on the current set of feature values, the Shapley value illustrates how an individual feature contributes to the difference between the actual and average predictions(Winter 2002, Lundberg, Nair et al. 2018).

### 2.5 Models for comparison

Several classical machine learning methods have been employed to predict continuous variables based on a set of features, including support vector regression, random forest, xgboost, etc. In some previous studies(Srivastava, Eitel et al. 2019, Tamez-Pena, Orozco et al. 2019, Vang, Cao et al. 2019), these machine learning models were used to predict fluid intelligence scores by using artificially extracted brain morphological features. A comparison was made between these models and ours for predicting fluid intelligence scores. Multi-layer perceptron is also used as a baseline model to verify the effectiveness of using the reconstruction loss function of autoencoder as a constraint to predict the fluid intelligence score.

### 2.6 Experiment settings

As a result of the random division of training and test sets, 8073 people were selected for the training set, and 3461 people were selected for the test set. To calculate 95% confidence intervals, 100 bootstrapped samples were used to estimate model performance on the test set. The neurons in the hidden layer of the autoencoder were pre-set to 50, 100, 200, and 400. Using grid search results, it was found that a model with 100 neurons performed best in the validation set. Therefore, we set the number of neurons in the hidden layer at 100. We initialize the learning rate to 0.1, then it decays to 0.99 of the previous step every 5 epochs. Based on pre-experiments, we found that setting λ_*θ,*_ λ_*φ*_, and λ_*ψ*_ in the objective function to 0.1 produced the best validation set performance. Except for the decoder, the structure of the multilayer perceptron is the same as that of the autoencoder. During the training process, we adopted the approach of early stopping, and the early stopping point is the first time the validation set’s MSE increases. AE and MLP are implemented through the Pytorch platform. Classic machine learning algorithms in this research are implemented using the scikit-learn library.

### 2.7 Evaluation metrics

To evaluate the performance of AE and MLP, we use MSE as an evaluation metric. MSE is defined in statistics as the mean of the squared difference between the predicted value and the true value. It can be calculated by Eq. 2.

#(2) $$MSE=\frac{1}{N}\sum_{i=1}^{N}{\left(y-\hat{y}\right)}^{2}$$

where, N is the total number of subjects, *y* is the real intelligence score, and *ŷ* is the predicted score of the prediction model.

Meanwhile, we use Pearson correlation coefficient (PCC) to measure how close the predicted value is to the actual value. It has been widely used in previous research and is considered superior to MSE for the evaluation of model performance. In statistics, PCC represents the linear correlation between two variables, whose value range is between [−1,1]. The calculation is shown in Eq. 3.

#(3) $$PCC=\frac{\sum_{i=1}^{N}\left(x_{i}-\bar{x}\right)\left(y_{i}-\bar{y}\right)}{\sqrt{\sum_{i=1}^{N}{\left(x_{i}-\bar{x}\right)}^{2}}\sqrt{\sum_{i=1}^{N}{\left(y_{i}-\bar{y}\right)}^{2}}}$$

where, *x*_*i*_ and *y*_*i*_ represent the true and predicted values, respectively, and $\bar{x}$ and $\bar{y}$ represent their sample means.

In order to compare the differences in the PCC of the model between subjects of different genders, we used two independent samples t test to test the hypothesis of the difference in PCC. The calculation is shown in Eq. 4.

#(4) $$t=\frac{\left(r_{1}-r_{2}\right)}{\sqrt{\frac{s_{1}^{2}}{n_{1}}+\frac{s_{2}^{2}}{n_{2}}}}$$

where, *r*_1_ and *r*_2_ represent the pearson correlation coefficient between the predicted value and the real value of the model in different gender groups, *s*_1_ and *s*_2_ represent their standard deviations, and *n*_1_ and *n*_2_ represent the number of people of different genders.

## 3 Results

### 3.1 Basic information on the study population

**Figure 3:**
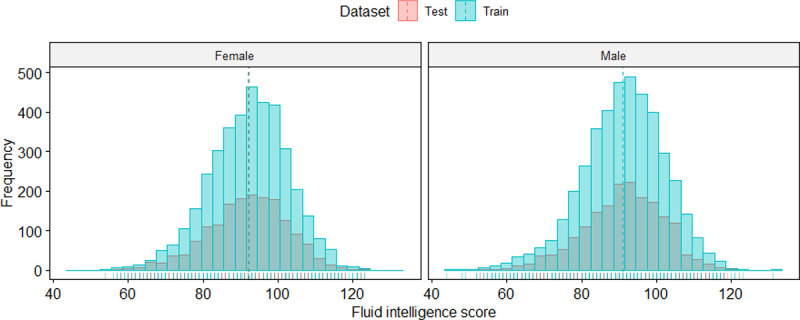
Distribution of Fluid Intelligence Scores in Samples of Different Genders.

The demographics of the 11,534 adolescents we studied are summarized in Table 1. There were 5504 males (47.7%) and 6030 females (52.3%) among them. The mean age of the objects interviewed was 130.47 ± 14.3 months. For all samples, the uncorrected mean fluid intelligence score was 91.6 ± 10.62. The demographic characteristics of the training and test samples are balanced. Figure 3 shows the distribution of uncorrected fluid intelligence scores among subjects of different genders.

### 3.2 Model performance

As shown in Table 2, our method achieves the best performance on the test set (MSE is 105.212 ± 2.53, PPC is 0.209 ± 0.02). In comparison with MLP, LR, RF, SCR, and Xgboost, our method improves MSE scores by 0.016, 0.041, 0.37, 0.04, and 0.085, respectively. The results show that our proposed method is significantly superior to classical machine learning models in predicting fluid intelligence scores. We then tested the performance of each model on male and female adolescent populations. Figure 5 illustrates the PCC of AE and MLP, while Fig. 6 shows the PCC of classical machine learning models. According to the results, all models performed better on female adolescents than on male adolescents.

### 3.3 Feature importance

To better understand which features drive predictions, we examine the feature importance of each individual model. We calculated the SHAP value of each feature for each sample in the training set separately for AE and MLP, then we took the absolute number of the SHAP value of each feature and calculated their average value in the population. Then we sorted the average value of the absolute value of SHAP of each feature from large to small. Table 3 summarizes the top 10 features and the mean absolute SHAP contained in the AE and MLP. Among the features listed in Table 3, sulcal depth of right hemisphere temporal pole, volume of left hemisphere temporal pole, sulcal depth of left hemisphere middle occipital gyrus, volume of left hemisphere middle temporal gyrus are jointly selected by AE, MLP. The AE model gave the highest priority to the sulcal depth of right hemisphere temporal pole, where an increase in depth correlated with an increase in fluid intelligence scores. Figure 7 shows in more detail the top 20 features selected by the AE model for predicting fluid intelligence. Figure 8 shows the relationship between the values of these 20 features and SHAP. We can see that not only the sulcal depth of right hemisphere temporal pole is positively correlated with the fluid intelligence score, but the volume of left hemisphere temporal pole and volume of left hemisphere anterior transverse collateral sulcus are also positively correlated with the fluid intelligence score.

## 4 Discussion

Fluid intelligence has been an established metric in psychology and education research since the early 70’s(Jensen 1974). In standard Gf assessments, multiple-choice questions are administered nonverbally(Raven 1983). There have been some studies exploring how functional MRI can predict the Gf score, but no study has examined whether structural T1-weighted MRI can predict the Gf score. Several studies have utilized 3D MRI images directly to predict fluid intelligence, however these methods have a higher prediction variance than classical machine learning models based on manual features. It may require a greater number of samples during the training phase if CNN is directly used to predict the fluid intelligence score(Li, Jiang et al. 2022). As a new approach to fluid intelligence score prediction, we propose to investigate whether individual brain anatomical properties, as revealed by T1-weighted MRI, can be used to predict fluid intelligence scores. Our study evaluates the performance of two deep learning models in predicting Gf in order to investigate this hypothesis.

**Figure 4:**
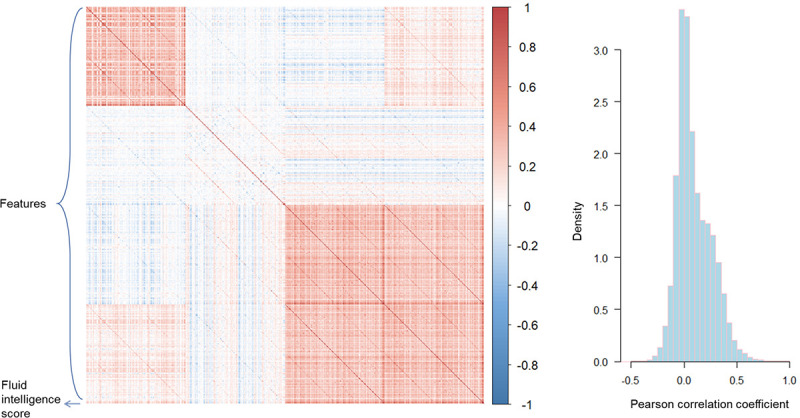
Correlation Matrix between Traits and Fluid Intelligence Scores

**Figure 5:**
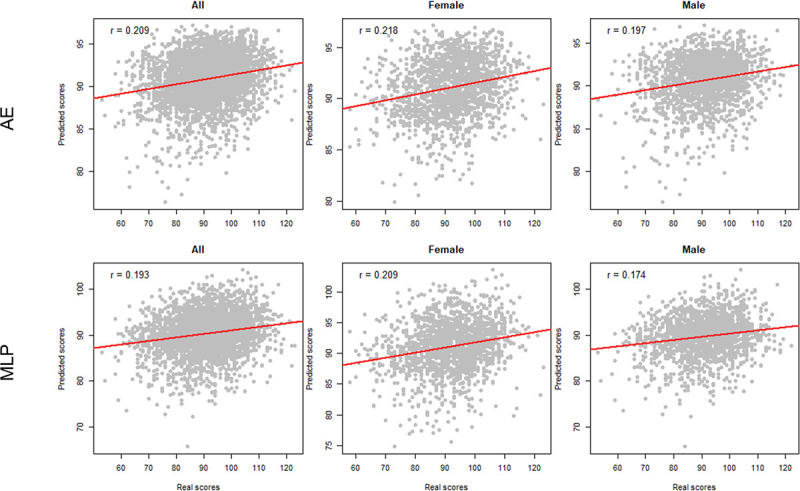
Scatter Plots for AE and MLP in Different Populations within the Test Set. AE: Autoencoder; MLP: Multilayer Perceptron.

**Figure 6:**
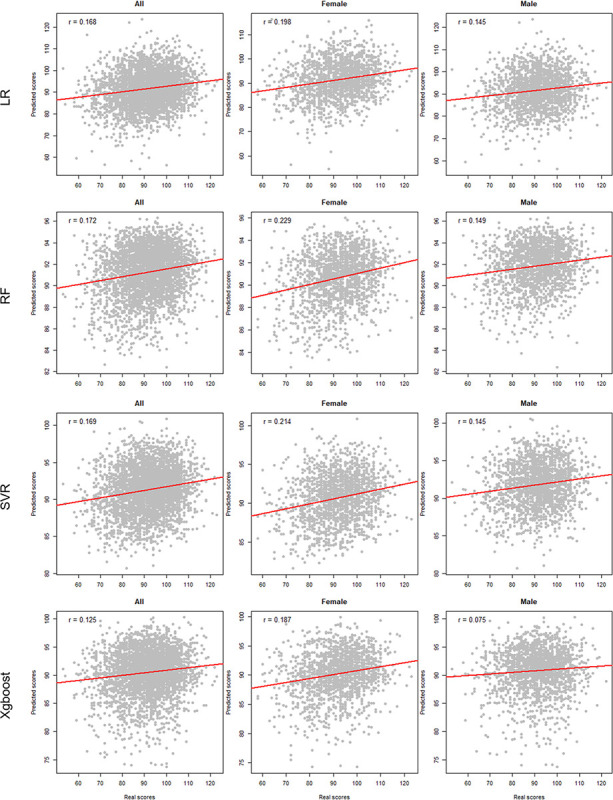
Scatter Plots for Classical Machine Learning Models within the Test Set. LR: Logistic Regression; RF: Random Forest; SVR: Support Vector Regression; Xgboost: Extreme Gradient Boosting.

**Figure 7:**
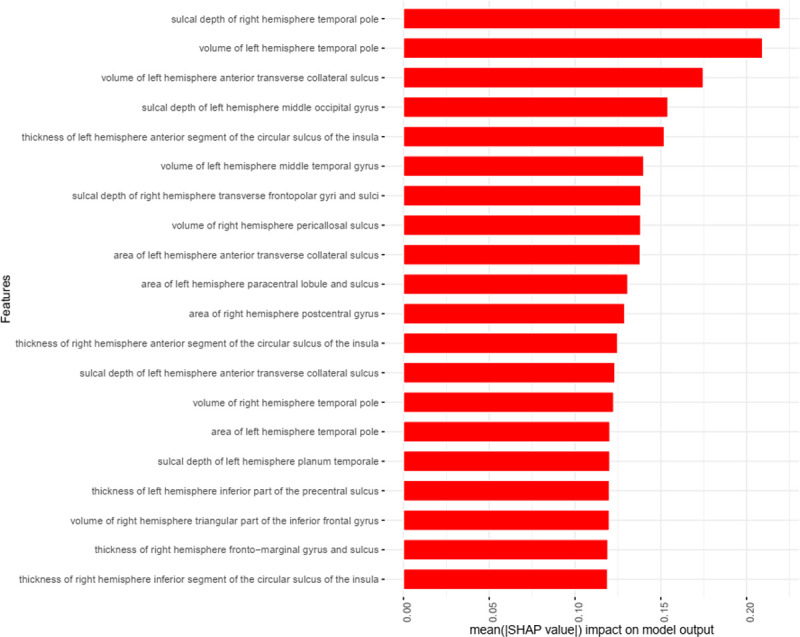
Top 20 Features Sorted by Mean Absolute SHAP Value for AE.

**Figure 8:**
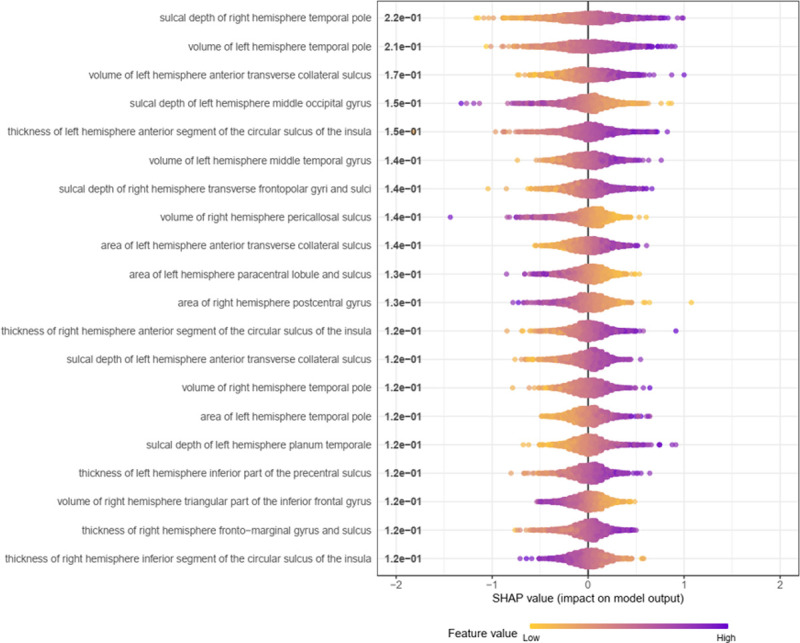
SHAP Values of the Top 20 Features for Each Subject in the Training Data. For each subject, SHAP values are plotted horizontally, stacking them vertically to prevent overlap. The color of each dot depends on the value of that feature, from low (yellow) to high (purple). In this example, the feature will appear smooth if its impact on the model’s prediction changes smoothly as its value changes.

Our motivation for using AE to predict fluid intelligence scores is its ability to compress highly dimensional features. Furthermore, because neural networks can approximate nonlinear functions, the regression branch in our proposed model can successfully learn the nonlinear relationship between the morphological features of the cortex and fluid intelligence scores. In this paper, our core contribution is to use AE to reconstruct features and use the reconstruction loss as an additional constraint to assist regression branch prediction. Results from this approach appear to be successful. To avoid overfitting problems in MLP, regular terms of L1 and L2 norms are often used in previous studies. Moreover, the dropout layer has also been demonstrated to be effective in preventing the problem of overfitting. Unfortunately, these methods cannot make the model learn the relationship between features. It can be seen from Fig. 4 that there is a correlation between the morphological features of the brain, and the Pearson correlation coefficient between 12.4% of the feature pairs is greater than 0.3. These correlations have a strong impact on the extrapolation of the model. As neural networks extract features adaptively, their purpose is determined by their objective function. In MLP, the model only needs to capture the relationship between features and fluid intelligence scores when adaptively extracting features. But in AE, the model needs to take into account the correlation between features when adaptively extracting features. As a result of this additional constraint, the model is able to be generalized more effectively. Our experimental results demonstrate this, where AE outperforms MLP on the test set.

Based on previous observations of sex differences in the growth and maturation of different brain structures in child and adolescent subjects(Sowell, Trauner et al. 2002, Giedd and Rapoport 2010), we paid special attention to differences in the predictive power of the model between sexes. According to the results of the t test, we found that whether in AE or in MLP, the PCC predicted by the model for female adolescents was always better than that for male adolescents (P < 0.001, P < 0.001). This result is consistent with that of previous studies(Ranjbar, Singleton et al. 2019). From Fig. 3, we can see that the distribution of fluid intelligence scores between different genders is roughly the same. Therefore, we analyzed that the reason for the significant difference in PCC between different sexes may be the sex difference in the underlying mechanism of fluid intelligence in adolescent brain development. Therefore, we further analyzed the feature importance of the AE model in different gender objects.

However, Table 4 shows that there are differences in the distribution of important features selected by the AE model in different gender groups. The three characteristics of right hemisphere temporal pole depth, left hemisphere temporal pole volume, and left hemisphere anterior transverse collateral sulcus volume are the top three across genders. When it comes to characteristics after the fourth place, however, the distribution is different between the sexes. Differences in the ranking of feature importance in developing brains may be due to sex differences in the mechanisms underlying fluid intelligence. A further study is needed to assess the radiographic predictors of Gf in developing young males and females.

Furthermore, we note that the variance of the predicted values is smaller than that of the true values, indicating that the distribution of the predicted values is significantly narrower than the true values. The reason for this phenomenon in our analysis is that these characteristics can only explain part of the attribution of fluid intelligence, so these predictions will be tightly clustered together. Clearly, more information is needed to better understand the relationship between structural imaging and fluid intelligence.

## 5 Conclusion

We constructed a weak but stable correlation between brain structural features and raw fluid intelligence using autoencoders. The results of our analysis indicate that predicting fluid intelligence solely on the basis of these morphological characteristics proves to be challenging. Despite this, our results indicate that incorporating reconstruction loss into deep learning models can improve the generalization of deep learning models in predicting fluid intelligence scores. In order to improve the predictive performance of fluid intelligence based on neuroimaging features, future research should explore ensemble regression strategies utilizing multiple machine learning algorithms. Additionally, we discovered that the distribution of feature importance for predicting fluid intelligence scores for adolescent populations differed across genders. This evidence suggests a need for further investigation of the relationship between structural imaging and fluid intelligence.

## Figures and Tables

**Table 1:** Summary of Demographic Characteristics of the Sample

		Overall	Train	Test	P value
N		11534	8073	3461	
Age (mean (SD))		130.47 (14.30)	130.34 (14.25)	130.80 (14.42)	0.110
Gender(n %)	Female	5504 (47.7)	3841 (47.6)	1663 (48.0)	0.657
	Male	6030 (52.3)	4232 (52.4)	1798 (52.0)	
Uncorrected Fluid Intelligence Scores (Mean (SD))		91.60 (10.62)	91.65 (10.69)	91.51 (10.45)	0.528
Family Income (n %)	<$25,000	1576 (14.9)	1118 (15.1)	458 (14.5)	0.490
	$25,000-$49,999	1533 (14.5)	1052 (14.2)	481 (15.2)	
	$50,000-$99,999	2997 (28.4)	2114 (28.6)	883 (28.0)	
	>$100,000	4453 (42.2)	3117 (42.1)	1336 (42.3)	
Parents’ Educational Level(n %)	High School/GED or Less	1645 (14.3)	1158 (14.4)	487 (14.1)	0.704
	Some College or Associate’s Degree/Bachelor’s Degree	5932 (51.5)	4133 (51.2)	1799 (52.1)	
	Greater than Bachelor’s	3944 (34.2)	2776 (34.4)	1168 (33.8)	

SD: Standard Deviation

**Table 2:** Performance of Different Models in the Train Set and Test Set

	Train set		Test set	
	PCC	MSE	PCC	MSE
AE	0.211 ±0.02	87.334 ± 2.25	0.209 ± 0.02	105.212 ± 2.53
MLP	0.199 ± 0.02	92.662 ± 2.32	0.193 ± 0.02	111.287 ± 2.58
LR	0.171 ±0.02	103.362 ± 3.21	0.168 ± 0.02	114.805 ± 3.50
RF	0.177 ± 0.02	95.023 ± 2.61	0.172 ± 0.02	107.150 ± 2.68
SVR	0.173 ± 0.02	98.477 ± 2.63	0.169 ± 0.02	108.108 ± 2.79
Xgboost	0.130 ± 0.02	101.716 ± 2.44	0.124 ± 0.03	113.330 ± 2.47

AE: Autoencoder; MLP: Multilayer Perceptron; LR: Logistic Regression; RF: Random Forest; SVR: Support Vector Regression; Xgboost: Extreme Gradient Boosting.

**Table 3:** The Mean Absolute SHAP of the Top 10 Features Selected by AE and MLP.

AE	Feature	Mean
		| SHAP |
	Sulcal depth of right hemisphere temporal pole	0.220
	Volume of left hemisphere temporal pole	0.209
	Volume of left hemisphere anteriortransverse collateral sulcus	0.175
	Sulcal depth of left hemisphere middle occipital gyrus	0.154
	Thickness of left hemisphere anterior segment of the circular sulcus of the insula	0.152
	Volume of left hemisphere middle temporal gyrus	0.140
	Sulcal depth of right hemisphere transverse frontopolar gyri and sulci	0.138
	Volume of right hemisphere pericallosal sulcus	0.138
	Area of left hemisphere anterior transverse collateral sulcus	0.138
	Area of left hemisphere paracentral lobule and sulcus	0.131
MLP	Sulcal depth of right hemisphere temporal pole	0.285
	Sulcal depth of right hemisphere superior frontal sulcus	0.282
	Sulcal depth of left hemisphere middle occipital gyrus	0.276
	Thickness of right hemisphere calcarine sulcus	0.262
	Volume of left hemisphere middle temporal gyrus	0.258
	Sulcal depth of right hemisphere lingual gyrus	0.255
	Area of right hemisphere anterior part of the cingulate gyrus and sulcus	0.246
	Sulcal depth of left hemisphere medial orbital sulcus	0.245
	Volume of left hemisphere temporal pole	0.241
	Thickness of right hemisphere superior frontal gyrus	0.240

**Table 4:** The Mean Absolute SHAP of the Top 10 Features Selected by AE in Objects of Different Genders

Male	Feature	Mean
		| SHAP |
	Sulcal depth of right hemisphere temporal pole	0.226
	Volume of left hemisphere temporal pole	0.195
	Volume of left hemisphere anterior transverse collateral sulcus	0.171
	Thickness of left hemisphere anterior segment of the circular sulcus of the insula	0.152
	Sulcal depth of left hemisphere middle occipital gyrus	0.148
	Sulcal depth of right hemisphere transverse frontopolar gyri and sulci	0.142
	Volume of left hemisphere middle temporal gyrus	0.139
	Volume of right hemisphere pericallosal sulcus	0.138
	Area of left hemisphere anterior transverse collateral sulcus	0.135
	Area of left hemisphere paracentral lobule and sulcus	0.130
Female	Sulcal depth of right hemisphere temporal pole	0.227
	Volume of left hemisphere temporal pole	0.204
	Volume of left hemisphere anterior transverse collateral sulcus	0.184
	Sulcal depth of left hemisphere middle occipital gyrus	0.157
	Thickness of left hemisphere anterior segment of the circular sulcus of the insula	0.146
	Area of left hemisphere anterior transverse collateral sulcus	0.144
	Volume of left hemisphere middle temporal gyrus	0.139
	Volume of right hemisphere pericallosal sulcus	0.135
	Area of right hemisphere postcentral gyrus	0.129
	Sulcal depth of right hemisphere transverse frontopolar gyri and sulci	0.127

## Data Availability

Data used in the preparation of this article were obtained from the Adolescent Brain Cognitive Development^SM^ (ABCD) Study (https://abcdstudy.org), held in the NIMH Data Archive (NDA). This is a multisite, longitudinal study designed to recruit more than 10,000 children aged 9–10 and follow them over 10 years into early adulthood. The ABCD Study^®^ is supported by the National Institutes of Health and additional federal partners under award numbers U01DA041048, U01DA050989, U01DA051016, U01DA041022, U01DA051018, U01DA051037, U01DA050987, U01DA041174, U01DA041106, U01DA041117, U01DA041028, U01DA041134, U01DA050988, U01DA051039, U01DA041156, U01DA041025, U01DA041120, U01DA051038, U01DA041148, U01DA041093, U01DA041089, U24DA041123, U24DA041147. A full list of supporters is available at https://abcdstudy.org/federal-partners.html. A listing of participating sites and a complete listing of the study investigators can be found at https://abcdstudy.org/consortium_members/. ABCD consortium investigators designed and implemented the study and/or provided data but did not necessarily participate in the analysis or writing of this report. This manuscript reflects the views of the authors and may not reflect the opinions or views of the NIH or ABCD consortium investigators.
